# Circle Diameter Impacts Stride Frequency and Forelimb Stance Duration at Various Gaits in Horses

**DOI:** 10.3390/s23094232

**Published:** 2023-04-24

**Authors:** Alyssa A. Logan, Alyson J. Snyder, Brian D. Nielsen

**Affiliations:** 1 School of Agriculture, Middle Tennessee State University, 314 W. Thompson Ln., Murfreesboro, TN 37129, USA; ajs2cj@mtmail.mtsu.edu; 2 Department of Animal Science, Michigan State University, 474 S. Shaw Ln., East Lansing, MI 48824, USA; bdn@msu.edu

**Keywords:** equine, horse, forelimb, circular, lunge, exercise, equestrian, asymmetry

## Abstract

The effects of gait and diameter have been studied independently, but rarely together in equine circular exercise studies. This study aimed to determine the impact of diameter (10-m or 15-m) at various gaits (walk, trot, and canter) on stride frequency or forelimb stance duration. Nine mature horses were outfitted with Tekscan™ Hoof Sensors on their forelimbs during circular and straight-line exercise at various gaits on a clay and sand arena surface. Statistical analysis was performed in SAS 9.4 with fixed effects of exercise type, recording, leg, and breed (PROC GLIMMIX, *p* < 0.05 significance). At walk (*p* < 0.0001) and trot (*p* < 0.001), stride frequency was lower during circular exercise. Stride frequency was similar between forelimbs at all gaits. At walk (*p* < 0.001) and canter (*p* = 0.01), stance duration was greatest during 10-m circle exercise. At walk (*p* = 0.0007), trot (*p* < 0.001), and canter (*p* < 0.0001), the inside forelimb had longer stance duration than the outside forelimb. Differences between forelimb stance durations may support asymmetrical travel while horses exercise on a circle at the walk, trot, and canter. These results demonstrate diameter and gait are important factors when evaluating forelimb kinematics during circular exercise.

## 1. Introduction

Circular exercise is used frequently in the equine industry under-saddle, in-hand via lunging, or in round pens. The motive for utilization of circular exercise varies widely, and may include warm-up procedures, early training techniques, advanced training techniques, rehabilitation, competition, lameness diagnostics and even arena space limitations. Requiring horses to trot in a circle on a lunge line is a common practice in lameness evaluations, as it increases the expression of many types of lameness and asymmetries [1].

When quadrupeds, such as the horse, travel in a circular fashion, they will lean into a circle [2,3]. As speed of travel around a circle increases, the lean-in angle of the body increases, as does the tilt of the limbs underneath the body [3,4]. A lean-in angle of up to 20° has been observed in horses trotting while on a lunge line [5]. Horses lean-in while traveling along a curve or through a circle to maintain upright balance, and likely align the limbs underneath themselves to avoid unnecessary joint torque [4]. Leaning into a turn results in greater peak vertical force on the outside limbs that push the horse around the circle, while the inside forelimb provides support [1]. In kinematic studies, further support has been found that the outside limbs have a pushing function during circular exercise, demonstrated by increased peak forces in outside limbs [6,7]. While horses are leaning in towards the center of a circle, the stance phase of the inside limb has been found to be greater than the outside limb in lunging and in galloping under saddle [1,7]. This aligns with findings that the outside leg will travel greater distance around a curve than the inside leg, and the outside leg will have a longer duty factor (percentage of stride duration that the limb is loaded) than the inside leg does [3].

Frequent and varied use of circular exercise, coupled with a recognition of asymmetrical forces between inside and outside limbs, has sparked interest in research on the mechanics and effects of equine circular exercise. In particular, the impact of environmental effects such as rider, tack, gait, speed, diameter, and footing. The Tekscan™ Hoof System (Tekscan Inc., Boston, MA, USA) has previously been used to measure loading parameters such as area and normal force and is reliable within a session of exercise [8,9,10,11,12]. While utilizing this system, it has been reported that the outside forelimb has greater mean area loaded at the canter than at the walk and trot when horses are exercised in a round-pen, suggesting the importance of evaluating varying gaits when studying circular exercise [12].

This study aimed to determine how the diameter used during circular exercise impacts stride frequency and forelimb stance duration at the walk, trot, and canter. In terms of circle diameter, it was hypothesized that within each gait the evaluated measurements of stride frequency and stance duration would vary between circle diameters. Based on previous reports, it was hypothesized that differences in stance duration between inside and outside legs would be evident during circular exercise, with the inside forelimb having longer stance duration than the outside. The Tekscan™ Hoof System was used to determine loaded area and force in the current study, with the results and methods related to loaded area and force published in a separate publication [12]. Stride frequency and stance duration, as reported in this article, were collected with data from the Tekscan™ Hoof System.

## 2. Materials and Methods

### 2.1. Horses

Nine mature Arabian and Quarter Horse animals in western-style training were utilized in this study (14 ± 2 years, 4 mares and 5 geldings). Animal procurement and involvement has previously been published [12]. All horses utilized in this study were evaluated one week prior to data collection by two board-certified veterinarians (large animal surgery and one also boarded in equine sports medicine and rehabilitation) with a Lameness Locator^®^ as well as subjective lameness evaluation. All horses were evaluated to have an American Association of Equine Practitioners lameness grade of <2 on each front limb.

### 2.2. Exercise and Sensors

The Tekscan™ Hoof System paired with glue-on shoes (SoundHorse Technology, Unionville, PA, USA) was used for data collection. Each horse wore a Tekscan™ Hoof Sensor on each forelimb, adhered by a glue-on shoe. Shoe and sensor preparation and application methodology have previously been published [8,12].

When using a sensor system such as the Tekscan™ Hoof System, calculating the average of multiple recordings (runs) of an exercise test is suggested, instead of reporting only one exercise test per situation [9,11,13]. Exercise included three recordings of each individual horse lead in a straight line at the walk and trot by a handler, and three recordings of each horse exercised in a small diameter (10 m) circle and large diameter (15 m) circle at the walk, trot, and canter. During each recording, there was a target of at least 10 strides. Straight-line exercise was recorded for 25 m in length for each recording. All circular exercise took place tracking counterclockwise within a non-banked round pen. Both circular exercise and straight-line exercise took place in the same arena, providing the same footing (clay and sand surface) between exercise types. Only one direction of circular exercise was evaluated so one limb would be consistently evaluated as the outside limb, and the other as the inside limb. The right limb was the outside limb, and the left limb was the inside limb.

Each horse began exercise recordings with straight line exercise, then were randomly assigned to perform small or large circle diameter exercise first, followed by the remaining circular exercise diameter. The canter was not performed on a straight line, due to handler and horse safety concerns. At the canter, all horses were on the correct, left lead for all recordings. Gait speed was purposefully not controlled between animals, as other gait analysis studies have supported allowance of animals to travel at their preferred natural speed within a gait [9,14,15,16,17]. During circular exercise, forward movement through gaits was encouraged by visual and auditory stimulation. The same experienced handler performed all straight line and circular exercise recordings to ameliorate handler differences.

Sensors were set to collect data at a rate of 112 frames/s for all conditions. Tekscan™ F-scan Software (version 6.85) was used to record and analyze exercise data. The first and last steps during each exercise recording were omitted in data analysis to ensure no transitional steps between gaits were included. Stance duration for the right or left forelimb were determined in Tekscan™ F-scan Software by recording how many frames a hoof was loaded for, then dividing that value by 112 frames to yield the stance duration for each stride in seconds. The beginning of a stance phase was determined when the first sensor cell was loaded, while the end of a stance phase was determined when the last sensor cell was loaded. Right and left leg stride frequency were calculated by recording the total amount of frames during a recording, which was then divided by 112 frames to yield the total amount of seconds of the recording. To yield stride frequency calculated as strides/s, the total right or left forelimb steps during the recording was divided by the duration (seconds) of the recording.

### 2.3. Data Analysis

Data were exported from the F-Scan Software and imported into SAS (9.4) for statistical analysis. At each studied gait (walk, trot, and canter), stride frequency and stance duration were evaluated with PROC GLIMMIX with main effects of exercise type (small circle, large circle, straight-line), exercise recording, leg, and breed, along with the interaction between exercise type and leg. Horse was included as a random effect in the model.

Breed was not a significant effect for stride frequency or stance duration at any gait. All results are presented as averages of all horses ± standard error of the mean (SEM). Statistical significance was set at *p* < 0.05, with trends set at *p* < 0.10.

## 3. Results

### 3.1. Walk

Stride frequency was not impacted by interaction of leg and type of exercise (*p* = 0.94). Both type of exercise (*p* < 0.0001) and the exercise recording (*p* = 0.01) were significant effects (Table 1).

Stance duration was different among exercise types (*p* < 0.001) and exercise recordings (*p* = 0.0019). The left (inside) leg had greater stance duration than the right leg (*p* = 0.0007) and the interaction between exercise type and leg was significant (*p* = 0.05, Table 2).

### 3.2. Trot

Stride frequency was not impacted by the interaction between exercise type and leg (*p* = 0.55). Both exercise type (*p* < 0.001) and recording (*p* = 0.05) were significant effects (Table 3).

Stance duration tended to be different between exercise types (*p* = 0.09, Table 3). Recording (*p* < 0.001, Table 3) and leg (*p* < 0.001) were significant effects, with the left (inside) leg having greater stance duration. There was a significant interaction between exercise type and leg (*p* < 0.001, Table 4)

### 3.3. Canter

Type of exercise (*p* = 0.38) and exercise recording (*p* = 0.14) were not significant effects for stride frequency. The interaction between exercise type and leg was not significant (*p* = 0.30).

Exercise type (*p* = 0.01) was a significant effect for stance duration (Table 5), while exercise recording was not (*p* = 0.12, Table 5). Leg was a significant effect (*p* < 0.0001), with greater stance duration on the left (inside, 0.21 s ± 0.01) leg compared to the right (outside, 0.20 s ± 0.01) leg. The interaction between exercise type and leg was not significant (*p* = 0.29).

## 4. Discussion

This study hypothesized that within the gaits of walk, trot, and canter, stride frequency and stance duration would vary between circle diameters. This was partially proven, as stride frequency and stance duration were both different between circle diameters at the walk. Stride frequency differed between circle diameters at the trot, and stance duration differed between circle diameters at the canter.

This study had a secondary hypothesis that differences in stance duration between inside and outside legs would be evident during circular exercise. At both the walk and trot, within circular exercise, the left (inside leg) had a greater stance duration than the outside leg. At the canter, there was no interaction between exercise type and leg. Given the lack of straight-line exercise performed at the canter, the difference between the left and right leg stance duration suggests that at both the small and large diameter circle the left leg had greater stance duration.

Forelimb loaded area, and normal force associated with this study have been published separately [12]. During circular exercise at the canter, it was found that the outside forelimb had greater mean area loaded than what was reported at the walk and the trot. The outside forelimb also had greater normal force during circular exercise at the canter compared to circular exercise at the trot. The loaded area and force results found gait had more impact to measured parameters than circle diameter. Gait and speed have been noted as important factors in circular exercise biomechanics in other studies as well [3,5]. However, circle diameter does impact factors such as carpal joint cartilage content, lean angle, and movement symmetry [5,18,19]. The reported results of stride frequency and stance duration in this study did reveal circle diameter to be an important factor within the gaits of walk, trot, and canter.

Stance duration appears to be affected more than stride frequency in the current study. A study evaluating kinetics of the forelimb while horses were lunged on an 8-m diameter at the trot in varying surfaces found stance duration to be longer for the inside forelimb, with no stride frequency difference between forelimbs [1]. When traveling around a curve, the outside legs may travel a longer distance than the inside legs [3]. The inside leg may have a greater stance duration to allow the horse to continue traveling around the curve, while maintaining propulsive forces [3]. For horses galloping on the correct (left) lead around a track, the left leg has been found to have longer stance duration than the right leg, on both a curve and a straight line [20]. At the trot on a 10-m diameter lunge circle, the inside leg has been found to have longer duty factor than the outside leg, suggesting longer stance duration [3]. Inclination of the third metacarpal has found to be greater on the inside leg than the outside leg on a 10-m lunge circle at the walk, trot, and canter as well [3]. This reflects that the inside limb adducts closer to the body during turning to aid in maintaining balance around a circle. In the current study, when exercising at the walk and trot, the inside, left leg had longer loaded stance duration.

It is interesting to note that this difference is only a trend when trotting a large circle, but there is a significant difference when trotting a small circle. This contributes to the hypothesis that a small circle incurs a greater degree of lean-in [5], with the potential for uneven loading between inside and outside legs, in greater effect than a large circle. The trot has previously been noted as a stable gait and is frequently used for lameness evaluations [21,22]. In the current study, stance duration was different between the large circle and the small circle at the walk and canter, with the small circle having greater stance duration. However, at the trot, the small circle and large circle were not different from each other. This may be due to the stability provided by the diagonal pairs in the trot [21,22].

The findings in this study are limited only to the forelimbs, and only to one bout of exercise, not long-term effects. When speed is controlled for in two-year-old race-trained Thoroughbreds, stride duration increased when galloping around a curve compared to galloping on a straight portion of tracks. However, when these same two-year-olds were evaluated as three-year-olds, stride duration was not different between running on curves or running on a straight portion of the track [7]. Duty factor, used to determine what percentage of the stride duration the limb was loaded, was not evaluated in the referred study. The increase in stride duration during the curve running found in the two-year-old cohort is most likely due to an increase in the stance duration. These results show that over time, an adaptation to curve running may be possible for galloping Thoroughbred horses in race training.

While the hind limbs were not evaluated in this study, the authors would expect to find an effect of circle diameter on hindlimb stride frequency and stance duration measurements as well. Studies evaluating the effects of circular exercise on hindlimb measurements report differences between inside and outside limbs and thoracic symmetry similar to the patterns described earlier [3,17,19]. One study found that while trotting in a 10-m diameter lunge circle while in a bridle with the lunge line attached to the inside bit ring, there was asymmetry of pelvic movement, similar to that of a mild hindlimb lameness [17]. Given the current interest in detecting clinically important asymmetries during lameness evaluation, further studies with the Tekscan™ Hoof Sensor System should include hindlimb measurements.

Utilization of loading data, such as force and area, coupled with stride timing measurements, can be performed with sensor systems such as the Tekscan™ Hoof System. The Tekscan™ Hoof System is found to be reliable within a session of exercise when adhered to the forelimbs with a glue-on shoe [8]. Differences between recordings found in this study may be attributed to varying number of strides represented in each recording. Within each recording, a target of 10 strides were recorded. However, a delay between the sensor datalogger and the “stop/start” trigger on the laptop may exist, allowing for more than 10 strides to be collected. This system is not suggested for use with one recording for research methods, but needs further work to determine how the variability between recordings can be reduced.

One limitation of this study is the lack of straight-line exercise recordings at the canter. This most likely contributed to a lack of exercise type and leg interaction at the canter. At both the walk and trot, the interaction of exercise type and leg showed no difference between forelimbs on a straight-line, but did find important differences between inside and outside limbs during circular exercise. Galloping around a curve on either the right or wrong lead can impact forelimb stride characteristics. Horses on the correct lead had longer stance duration on the inside leg than the outside leg, but horses on the incorrect lead had no difference between the inside and outside leg stance duration [20]. A circular exercise study including canter recordings at both leads on a straight line would be desired for better comparisons of straight-line exercise and circular exercise. It is worth noting that this study only evaluated circular travel to the left, and not the right. Potential preferences for the left or right direction may exist in this cohort of horses, but were not evaluated. The authors believed that sensors may not last for double the study duration for each horse to record both the right and left directions. In order to compare between studies utilizing a racing cohort, the authors believed the left direction was preferrable for this study.

This study also did not evaluate duty factor, as similar gait analysis studies have [3,20]. The current Tekscan™ Hoof System has not yet been used to determine duty factor when used on horses traveling at the walk, trot, and canter during circular exercise on arena surface. Given the nature of arena surface (clay and soil) to interact with hooves and sensors attached to hooves, the moment of unloading may be difficult to determine to calculate stride duration. Future studies are needed to determine the reliability of duty factor as calculated with the Tekscan™ Hoof System.

## 5. Conclusions

Overall, while studying stride frequency and stance duration, both gait and circle diameter are important factors. Within the small and large-diameter circles at the walk and trot, the left (inside) leg was loaded with longer duration. At the canter, stance duration was longer during small circles compared to large circles. Stride frequency was lower in small and large circles at the trot and walk compared to straight-line travel. These results contribute to the understanding of the biomechanics of equine circular exercise. Further understanding of how horses navigate turns of varying diameter and gaits is necessary to determine the recommendations for how circular exercise should be performed in training and competition.

## Figures and Tables

**Table 1:** Stride frequency and stance duration for exercise type and recordings at the walk.

Exercise Type	*n* (Steps)	Stride Frequency (Strides/s)	Stance Duration (s)
Straight	900	0.85 ^a^ ± 0.02	0.80 ^b^ ± 0.02
Large circle	757		
		0.82 ^b^ ± 0.02	0.81 ^b^ ± 0.02
Small circle		0.78 ^c^ ± 0.02	0.85 ^a^ ± 0.02
	638		
**Exercise recording**			
1	750	0.81 ^e^ ± 0.02	0.83 ^a^ ± 0.02
2	759	0.82 ^d^ ± 0.02	0.81 ^b^ ± 0.02
3	789	0.81 ^e^ ± 0.02	0.82 ^ab^ ± 0.02

^a,b,c^ Means lacking common superscript between exercise type or recording differ (*p* < 0.001). ^d,e^ Means lacking common superscript within recording differ (*p* = 0.01).

**Table 2:** Interaction between exercise type and leg for stance duration results at the walk.

Exercise Type	*n* (Steps)	Leg	Stance Duration (s)
Straight	450	Inside	0.802
	450	Outside	0.802
Large circle *	378	Inside	0.825
	379	Outside	0.800
Small circle *	319	Inside	0.859
	319	Outside	0.839
SEM			0.020

* Denotes left and right limb within an exercise type have different stance duration (*p* < 0.05).

**Table 3:** Stride frequency and stance duration for exercise type and recordings at the trot.

Exercise Type	*n* (Steps)	Stride Frequency (Strides/s)	Stance Duration (s)
Straight	692	1.43 ^a^ ± 0.02	0.357 ^y^ ± 0.013
Large circle	769		
		1.34 ^b^ ± 0.02	0.361 ^xy^ ± 0.013
Small circle		1.35 ^b^ ± 0.02	0.365 ^x^ ± 0.013
	708		
**Exercise recording**			
1	681	1.37 ^cd^ ± 0.02	0.368 ^a^ ± 0.013
2	790	1.36 ^d^ ± 0.02	0.364 ^a^ ± 0.013
3	698	1.38 ^c^ ± 0.02	0.350 ^b^ ± 0.013

^a,b^ Means lacking common superscript between exercise type or recording differ (*p* < 0.001). ^c,d^ Means lacking common superscript between exercise type or recording differ (*p* = 0.05). ^x,y^ Means lacking common superscript between exercise type tend to differ (*p* = 0.09).

**Table 4:** Interaction between exercise type and leg for stance duration results at the trot.

Exercise Type	*n* (Steps)	Leg	Stance Duration (s)
Straight	348	Inside	0.356
	344	Outside	0.359
Large circle ^†^	393	Inside	0.365
	376	Outside	0.357
Small circle ***	357	Inside	0.387
	351	Outside	0.343
SEM			0.13

^†^ Denotes left and right limb within an exercise type tend to differ (*p* = 0.08). *** Denotes left and right limb within an exercise type are different (*p* < 0.0001).

**Table 5:** Stance duration within exercise type and recording at the canter.

Exercise Type	*n* (Steps)	Stance Duration (s)
Large circle	640	
		0.18 ^b^ ± 0.010
Small circle	596	0.19 ^a^ ± 0.010
**Exercise recording**		
1	387	0.21 ± 0.010
2	414	0.21 ± 0.010
3	435	0.20 ± 0.010

^a,b^ Means lacking common superscript within exercise type differ (*p* = 0.01).

## Data Availability

Datasets used and analyzed during the present study are included in the article. Raw data and further inquiries can be directed to the corresponding author.
